# A rare case of complete male genital self-amputation posing challenges in the psychiatric diagnosis and management

**DOI:** 10.1016/j.heliyon.2021.e07349

**Published:** 2021-06-18

**Authors:** Fotios Tsanakalis, Abdullah Almadhyan, Despina Flondell-Sité

**Affiliations:** a Department of Liaison Psychiatry and Psychiatric Emergency Cases, Skåne University Hospital, Malmö, Sweden; b Department of Urology, Skåne University Hospital, Malmö, Sweden

**Keywords:** Genital self-mutilation, Klingsor syndrome, GSM, Command hallucinations

## Abstract

Genital self-mutilation (GSM) is a rare phenomenon encountered mostly within the context of severe mental illness. The following case report highlights a rare case of self-inflicted total penile self-amputation in a patient with a psychiatric history of polydrug abuse and attention deficit disorder (ADD). The patient engaged in penile self-amputation under the influence of command hallucinations and religious delusions. He was operated on with microsurgical penile replantation but the penis had to be amputated after two weeks because of postoperative complications. The patient was admitted for compulsory psychiatric treatment. During the prolonged hospitalization course, he was arrested for stabbing two other patients and was transferred to a forensic psychiatric unit. The case fits the description for Klingsor Syndrome and involved multiple interacting risk factors that complicated the initial presentation and the ensuing management of the condition in the hospital setting.

## 1 Introduction

Intentional self-mutilation has been defined as the direct and deliberate physical self-injury without suicidal intentions [1]. Major self-mutilation (MSM), which involves major trauma and tissue damage, is rare and often results in persistent loss of an organ or its function [2]. Common organs involved in MSM are the eyes, the limbs, and the genitals.

Male genital self-mutilation (GSM) is one of the most dramatic and bizarre forms of MSM and is often encountered within the context of severe psychopathology. The most common underlying psychiatric disorders identified in a systematic review of 173 cases of male GSM were schizophrenia spectrum disorders (49%), substance use disorders (18.5%), personality disorders (15.9%), and gender dysphoria (15.3%) [3]. Substance use disorders and personality disorders were most commonly identified as secondary comorbidities complicating the presentation of GSM. The same review subcategorized the GSM cases in four injury subtypes (amputation, castration, mutilation, and combined amputation/castration) and demonstrated with statistical significance that self-amputation was most common in individuals diagnosed with schizophrenia spectrum disorders, while self-castration was found to be more common among cases with gender dysphoria.

GSM associated with religious delusions has been described by the term Klingsor Syndrome [4]. In Wagner's opera Parsifal, Klingsor was a fictitious character who castrated himself in order to remain chaste so that he could gain acceptance into the religious brotherhood of the Knights of the Grail [5]. Schweitzer proposed to expand the term Klingsor Syndrome so that it would include GSM resulting from all delusional syndromes [6].

Here we present a case report of a male patient who performed a complete penile self-amputation under the influence of command hallucinations and religious delusions.

## 2 Case report

### 2.1 Index event

**Figure 1:**
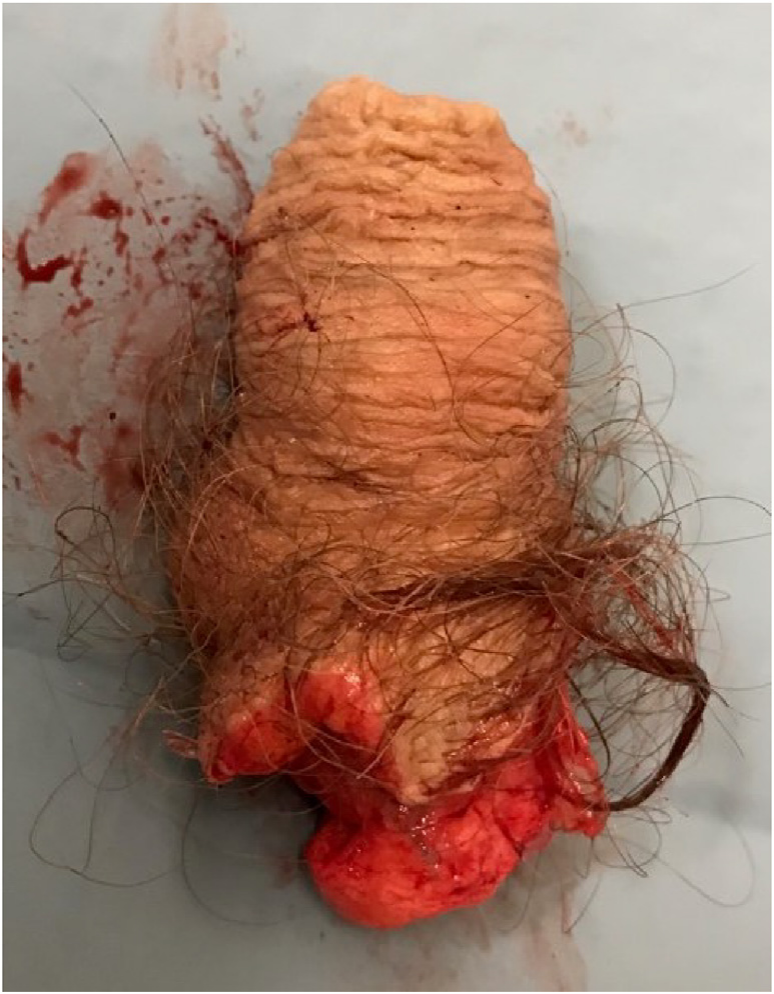
Distal amputated penis.

A Caucasian, male patient in his early fifties was brought to the Emergency Department by ambulance and with police escort. The patient was met by the police and the paramedics in the street naked, covered in blood deriving from the genital area and trying to cut his eyes out with a knife. He was deemed to pose an imminent risk to himself and others and was forced down to the ground by the police after a violent struggle that caused him bone fractures. The patient's amputated penis (Figure 1) was covered in a moist saline gauze and placed in a plastic bag which itself was kept in another plastic bag containing ice water.

**Figure 2:**
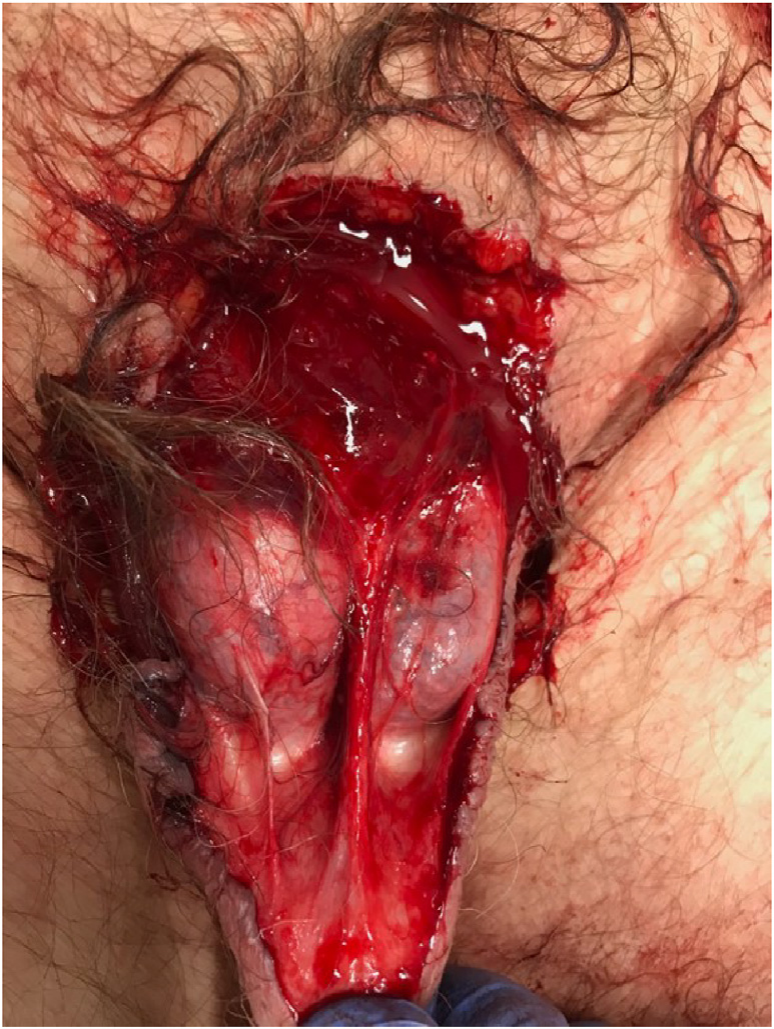
Image showing patient's injuries at the time of presentation. Self-inflicted complete penile amputation at the base of the penis and scrotal injury.

The physical examination revealed complete penis amputation, scrotal injury, and multiple cuts in both eyelids. The clean-cut penile incision was consistent with injury caused by a sharp knife. The scrotal injury involved a deep incised wound exposing but leaving uninjured both testicles (Figure 2). X-ray imaging demonstrated a right distal diaphyseal ulnar fracture and a left lateral clavicle fracture. The urine testing drug kit was positive only for cannabis (THC).

### 2.2 Past psychiatric history

The patient has been in contact with the psychiatric services for polysubstance abuse (cannabis, amphetamine, alcohol, opioids) and attention deficit disorder (ADD). His drugs of choice have been cannabis (since 13 years old) and amphetamine (since 18 years old). He was diagnosed with ADD in his early thirties and received intermittent treatment with methylphenidate 108 mg daily, but the medication was repeatedly stopped due to relapses to multidrug abuse. He was raised by parents with severe drug dependence and has a history of childhood abuse and neglect. When it comes to his educational background, he has only completed secondary school. He has been unemployed and living on welfare benefits during the longest period of his adulthood and has only held a few short-term manual occupations. He has a history of suspected drug-induced psychosis three years before the index event, which led to compulsory admission on seven occasions within six months, spending in total more than four months as an inpatient. The core of the patient's psychotic symptoms consisted of religious delusions related to the Day of Doom. During one of these admissions, he attempted teeth self-extraction acting upon command hallucinations. The urine toxicology test on every admission was positive for Cannabis. The methylphenidate treatment was paused during the admissions but restarted after every discharge to minimize the risk of relapse to amphetamine abuse. He was discharged with the diagnosis of drug-induced psychosis and continued treatment with methylphenidate and LAI (long-acting injectable) perphenazine decanoate for one year. During this period, he remained in remission. He then chose to discontinue his treatment against medical advice and a few months later relapsed in regular abuse of amphetamine. Two months before the index event he was put back on methylphenidate 108 mg daily. The staff of the patient's supported housing reported that he was exhibiting hallucinatory behavior and talking about demons a few days before the incident.

### 2.3 Hospitalization course

#### 2.3.1 Surgical management

The patient was sedated and intubated due to extreme aggression. A team of urologists and plastic surgeons were involved in the operation which took almost 5 h. The urethra, dorsal arteries, dorsal veins and nerves were anastomosed. The revascularisation was done 6.5 h after amputation and 3.5 h after arrival at the hospital. He was urgently operated with microsurgical penile replantation and transferred to the ICU (Intensive Care Unit) for close monitoring of the patient's agitation upon wake-up. The patient exhibited violent and abusive behavior upon extubation and was therefore reintubated and sedated with Propofol and Dexmedetomidine in order to prevent harm to himself or the staff. On day 1 after replantation, the penis was spotted purple but warm. Enoxaparin and antibiotics were administered at the ward. Epidermolysis of the scrotal skin on the right side was observed on day 5. The glans penis was warm and had normal color but the shaft was dark blue. Discharge from the wound with Klebsiella bacterial growth was observed on day 9. On day 12, skin necrosis at the whole penile shaft was observed. Unfortunately, the penis became ischemic two weeks after reimplantation and had to be amputated.

#### 2.3.2 Psychiatric management

The consultant liaison psychiatrist was called to the ICU for assistance at the moment of the third attempt to wake-up on day 3 of the hospital course. Upon waking up, the patient was hostile, agitated, and identified himself as “Lucifer”. The mental health status examination revealed delusions of control from the Devil, passivity of volition, and suspected auditory command hallucinations. He exhibited inappropriate affect and poor insight. The patient was sectioned under the Swedish Compulsory Psychiatric Care Act and received compulsory treatment with haloperidol IV and zuclopenthixol acetate IM. He was then transferred to the inpatient urology clinic where he was put on one-to-one monitoring by psychiatric staff due to the risk for unpredictable psychotic behavior.

In follow-up psychiatric interviews, the patient revealed that, a few days before the incident, he started to experience auditory hallucinations with content related to the imminent end of the world. The voices, that he could identify as pertaining to “God” and “Lucifer”, were commanding him to perform specific tasks to prevent Doomsday. On the day of the penile self-amputation, he experienced that Lucifer's commands were getting so intense and threatening that he could no longer resist them. He was ordered to sacrifice an acquaintance of his and complying with the auditory commands, he picked up a sharp, kitchen knife and went to his target's house. He broke into the building by breaking a window glass but did not find anybody present there. The patient revealed that he was then commanded by Lucifer to sacrifice his genitals in order to prevent the End of the World. Obeying to Lucifer's commands, the patient cut his penis and placed it in his mouth while also cutting his scrotum open. The command hallucination upon which he was acting when he was located and neutralized by the police, involved the self-enucleation of the eyes.

The patient initially claimed that he had abused methamphetamine, cannabis, and heroin before the incident but later on retracted this information and stated that he had intaken hallucinogenic mushrooms.

During the hospitalization course in the urology clinic, the patient continued to experience command hallucinations instructing him to injure himself and to kill himself by self-immolation. On one occasion he attempted to break one of his fingers by using a glass bottle but was hindered by the staff. On another occasion, he forcibly removed his urinary catheter and escaped from the ward running away naked complying with orders from the Devil. He was located in a nearby park within a few minutes and retrieved to the hospital but this time he was transferred to the psychiatric clinic as it was very hard to ensure the patient's safety in the urology ward. Antipsychotic treatment with high dose haloperidol (between 12-16 mg/day) per os and long-acting injectable (LAI) perphenazine decanoate (216 mg every 2 weeks) as an add-on was prescribed. The observational monitoring was later on downgraded from one-to-one to intermittent observation every 15 min as the patient gave the impression that he was doing better. A few weeks later, he stabbed two other patients in the ward with a kitchen knife. He assaulted the first patient while eating lunch in the ward's dining room so the staff managed to intervene in time. The second assault occurred during the night 8 days later when he entered the room of another sleeping patient and stabbed him in the face and neck. Despite the assaults, he was not put into solitary confinement and was not restrained physically as he was cooperative and did not exhibit any signs of agitation while in the ward except for these isolated incidents. The use of coercive measures, such as physical restraint and seclusion, is strictly regulated by the Swedish Compulsory Psychiatric Care Act and warranted only when it is unavoidable and to as little an extent as possible [7]. It later turned out that despite the initial impression of clinical improvement, the patient had continued to experience intermittent command hallucinations attributed to Lucifer. Due to the violent assaults, he was arrested and transferred to a secure forensic psychiatric unit. There he underwent a forensic psychiatric investigation including a risk assessment for recidivism in violent crime. The patient was diagnosed with schizophrenia (DSM-5 #295.90) and was considered as having a high risk for recidivism in severe criminality. He was sentenced to involuntary forensic psychiatric care with “special court supervision” (SCS), which means that he cannot be discharged without court approval due to the high risk for recidivism.

Informed consent was obtained from the patient for the publication of this case study after consent capacity clinical assessment at a timepoint during the hospitalization when he was temporarily stable and cooperative to voluntary health care.

## 3 Discussion

This case report presents a rare case of complete GSM in a patient with complex psychiatric comorbidity. Our case fits the criteria for Klingsor Syndrome as the penile self-amputation occurred under the influence of religious delusions and auditory command hallucinations.

Major self-mutilation of such severity is so rare that it is hard to predict unless there has been a previous attempt at self-injury, or the patient has spoken about intentions to remove or injure an organ [8]. As a matter of fact, our case subject had a previous history of attempted teeth self-extraction during an earlier episode of psychosis. The patient's claims of acute intoxication with amphetamines and opioids prior to the GSM were not corroborated by the multi-panel urine drug test kit. Nevertheless, the patient's history of psychoactive substance use posed a challenge in the process of psychiatric diagnosis, especially during the initial part of the hospitalization course. According to DSM-5, the persistence of psychotic symptoms for a substantial period of time (e.g., about one month) after the cessation of acute withdrawal of severe intoxication with a psychoactive substance could be used as evidence of an independent psychotic disorder [9]. In our case, the psychotic symptoms did not go into full remission despite inpatient antipsychotic treatment with two antipsychotics for more than two months. In fact, two months after the admission, the patient severely assaulted two other individuals in the psychiatric ward under the influence of homicidal command hallucinations. His experience that the GSM was carried out by the Devil taking control of his body can be classified as passivity of volition and passivity of impulse. Passivity experiences belong to the First Rank Symptoms (FRS) for schizophrenia and point towards a primary psychotic disorder.

The subject in our case study was experiencing command hallucinations consistent with delusional beliefs about the imminent End of the World and the fear of burning in hell. The voices pertained to God and Lucifer; figures regarded as omnipotent by our patient. The presence of delusions related to a perceived threat or an overriding of one's internal controls has been referred to as threat/control override (TCO) symptoms and is associated with increased risk for violent behavior [10]. The concurrence of command hallucinations with a consistent delusional belief and the conviction of the patient that the voices are omnipotent have been found to increase the likelihood of compliance [11]. It is also worthwhile to mention that delusions related to major self-mutilation have been found to be similar to those associated with many cases of psychotically motivated homicide, in that the patients were perceiving a threat to their own existence [12]. These findings have implications for risk assessment when evaluating a patient experiencing auditory hallucinations with violent content. It is recommended that patients who are found to be experiencing this constellation of alarming psychopathological symptoms after self-mutilation be placed on continuous one-to-one monitoring by experienced psychiatric staff to prevent further injury to themselves or others. Considering that the initial management of patients presenting with GSM takes place in a surgical/urological ward, it is imperative to strive for an integrated liaison-type psychiatric intervention as early as possible after presentation in order to improve the outcomes of the surgical intervention and also prevent further harm due to complications in the psychiatric manifestation.

In an effort to bring further clarity and understanding on the case presented, it is important to reflect on the role of specific risk factors from the patient's history that may be of relevance for the development of the psychopathology presented above and its dire outcomes. The individual in our case has had almost continuous cannabis abuse since the early age of 13 and regular amphetamine abuse since the age of 18. Additionally, he was raised in an abusive environment experiencing childhood maltreatment and neglect by parents with severe drug dependence. Cannabis exposure during adolescence conveys a higher risk for psychosis in later life and this risk has been found to be dose-related [13]. Several studies have demonstrated that the combined exposure to cannabis use and childhood trauma may increase the likelihood of developing psychotic symptoms to a greater extent than the risk expected for each individual environmental factor [14, 15, 16]. It is also of value to mention that a Danish, register-based study of 6,788 patients with substance-induced psychosis, demonstrated a 41.2 % conversion rate of cannabis-induced psychosis to schizophrenia [17]. The same study demonstrated that self-harm after a substance-induced psychosis was significantly linked to a higher risk of conversion. Our patient had been previously hospitalized for severe and persistent psychotic symptoms and received treatment with LAI but chose to discontinue his medication fifteen months before the index event and also relapsed in regular abuse of amphetamine. Two months before the index event, he was put back on high dosage methylphenidate (108 mg) without concurrent antipsychotic coverage and during ongoing daily cannabis abuse and sporadic amphetamine abuse. This is of particular significance as stimulants may have a detrimental effect across the spectrum of psychotic disorders and, along with cannabis, may play a causal role in some episodes of psychosis [18]. Stimulants may contribute to the burden of psychosis at all stages of illness, and cannabis use disorders are the strongest correlate of stimulant use disorders in studies of people with psychosis [19]. The synergetic pro-psychotic effects of cannabis and stimulants have been postulated to be exerted through the striatal D2→eCB→CB1 trans-synaptic pathway [20]. In hindsight, it is probable that our case subject had already converted to a chronic schizophrenia spectrum psychosis long before the index event but his substance abuse hindered the proper psychiatric diagnosis and treatment as his psychotic symptoms were deemed to be drug-induced. The cessation of the antipsychotic treatment with LAI, relapse into polydrug abuse, and later on the prescription of high dosage Methylphenidate may have all been factors tipping the patient “over the edge” to a recurrent psychotic episode with the devastating consequences of not only complete GSM but also attempted homicide.

## Declarations

### Author contribution statement

All authors listed have significantly contributed to the investigation, development and writing of this article.

### Funding statement

This research did not receive any specific grant from funding agencies in the public, commercial, or not-for-profit sectors.

### Data availability statement

The data that has been used is confidential.

### Declaration of interests statement

The authors declare no conflict of interest.

### Additional information

No additional information is available for this paper.
